# SEL1L3 suppresses colorectal cancer cell growth and metastasis by preventing endoplasmic reticulum-associated degradation of STING

**DOI:** 10.1038/s41419-026-08770-6

**Published:** 2026-05-03

**Authors:** Hui Zhang, Wenli Qian, Mengying Li, Xiuqun Zou, Xiaolin Chen, Keyan Miao, Mai Zhao, Mengjiang Xu, Jiali Dong, Jiamin Wang, Haixia Peng, Hao Jia, Zhaoyuan Hou

**Affiliations:** 1 Digestive Endoscopy Center & SKL-FMR, Tongren Hospital/Faculty of Basic Medicine, Shanghai Jiaotong University School of Medicine, Shanghai, China; 2 Yantai Stomatological Hospital/Peninsula Cancer Research Center, School of Stomatology, Shandong Medical & Pharmaceutical University, Yantai, China; 3 Shanghai Key Laboratory for Tumor Microenvironment and Inflammation, Department of Biochemistry & Molecular Cellular Biology, Shanghai Jiaotong University School of Medicine, Shanghai, China; 4 Department of Laboratory Medicine, Shanghai General Hospital, Shanghai Jiaotong University School of Medicine, Shanghai, China; 5 Department of Oral and Maxillofacial-Head and Neck Oncology, Shanghai Ninth People’s Hospital, Shanghai Jiaotong University School of Medicine, Shanghai, China; 6 Trauma Center, Shanghai General Hospital, Shanghai Jiaotong University School of Medicine Shanghai, Shanghai, China; 7 Shandong Key Lab of Complex Medical Intelligence and Aging, Shandong Medical & Pharmaceutical University, Yantai, China

**Keywords:** Tumour-suppressor proteins, ER-associated degradation

## Abstract

Endoplasmic reticulum associated degradation (ERAD) plays pivotal role in protein homeostasis and quality control in normal and cancer cells, yet the regulatory mechanism of ERAD remains elusive, especially regarding its ubiquitination function mediated by hydroxymethylglutaryl reductase degradation protein 1 (HRD1). Here, we report that Sel-1 Suppressor of Lin-12-Like 3 (SEL1L3) protein resided on the ER membrane can effectively prevent HRD1-mediated ERAD process via dual mechanisms: SEL1L3 disrupts SEL1L-HRD1 complex by mutually exclusively interacting with SEL1L and HRD1 respectively, resulting in concomitant prevention of substrate degradation; on the other hand, SEL1L3 can accelerate HRD1 protein degradation. Biologically, SEL1L3 inhibits colorectal cancer (CRC) cell growth and migration, which counteracts the oncogenic activity of HRD1; moreover, we identify STING as a HRD1 substrate and a critical downstream effector mediating tumor suppression activity of SEL1L3. Collectively, these data demonstrate that SEL1L3 is a critical regulator of ERAD and exerts a potent tumor-suppressing function, and that the SEL1L3/HRD1/STING axis plays a crucial role in CRC growth and migration.

## Introduction

The endoplasmic reticulum (ER) is the largest subcellular organelle and performs numerous functions, including protein synthesis and folding [1], lipid synthesis [2], and the maintenance of cellular calcium homeostasis [3]. Environmental factors, such as hypoxia, nutrient deprivation, reactive oxygen species (ROS) accumulation, low pH, and disruption of calcium balance, can adversely interfere with ER protein folding activity, thereby trigger endoplasmic reticulum stress (ERS) [4–8]. When the accumulation of unfolded and misfolded proteins in the ER exceeds a certain threshold, it triggers the unfolded protein response (UPR), which is a cellular defense mechanism designed to restore ER homeostasis or trigger cell death [9].

In eukaryotes, approximately 30% of all newly synthesized proteins pass through the ER, where they undergo folding and maturation [10]. As a fact, a fraction of nascent proteins inevitably fail to fold properly, as many of these proteins are ultimately disposed by a quality-control process known as ER-associated degradation (ERAD) [11–14]. The protein quality control process involves several steps, including recognition of misfolded substrates in the ER lumen and membrane, translocating them back into the cytosol (retro-translocation), interacting with ubiquitin and delivering substrates to proteasome for final degradation [15–17]. Each step of ERAD is tightly regulated by multiple ER chaperones [15–18]. However, growing evidence indicates that ERAD is implicated in the development of various diseases [12, 13, 19–22], our understanding of its machinery and the pathogenic importance of its underlying mechanisms remain limited.

The ERAD pathway operates through various parallel branches, organized around distinct membrane-resident E3 ubiquitin ligases [12, 23]. Hydroxymethylglutaryl reductase degradation protein 1 (HRD1) is a principal ER-resident E3 ligase [24] forming a complex with ER-resident single-transmembrane protein Suppressor/Enhancer of Lin-12-like (SEL1L) in mammals [25, 26], which is responsible for the degradation of a subset of misfolded and normal proteins in the ER [18, 27–30]. The HRD1 complex was initially discovered in yeast as responsible for the degradation of 3-hydroxy-3-methylglutaryl-CoA (HMG-CoA) reductase [31] and in *C. elegans* through genetic interactions with Notch [32]. Recently, studies have shown that activated STING is negatively regulated by the HRD1-SEL1L ERAD machinery, which has been demonstrated to control STING-mediated innate immunity and inflammation by ubiquitination-dependent degradation of STING [33, 34]. In addition to catalyzing the proteasome-mediated degradation of misfolded proteins in the ER [18, 27, 28], HRD1 can also function in a context-dependent manner by selectively degrading distinct substrates in the cytoplasm [35–37]. As such, HRD1 deficiency has been shown to cause diverse biological outcomes, including tumor suppression [38, 39], murine embryonic lethality [40, 41] and compromised T cell activation and B cell-mediated immunity [42].

Beyond the key ubiquitin ligase activities, other core components of the ERAD modules also play essential roles in degrading misfolded proteins. Among these components, SEL1L not only regulates the stability of HRD1 [26, 43, 44], but also is involved in substrate recruitment by interacting with lectins such as OS9 (Osteosarcoma amplified 9), XTP3-B (XTP3-Transactivated Gene B Protein), ERLEC1 and the E2 enzyme UBE2J1 to HRD1 [45–47]. SEL1L protein belongs to the SEL1L family, which comprises three members: SEL1L, SEL1L2 and SEL1L3. All SEL1L-family proteins share with C-terminal Sel1 repeats and single transmembrane domain, but differ in N-terminal effector domains and tissue distribution patterns [48]. The paralogous relationship between SEL1L and SEL1L3 suggests a potential function of SEL1L3 in ERAD process. Earlier research has identified SEL1L3 serves as a promising prognostic biomarker for atherosclerosis, colorectal cancer, melanoma, renal cell carcinoma and pulmonary carcinoid [49–52]. Recently, Chi-Ya et al. reported that SEL1L3 might exert tumor-suppressive effects in lung adenocarcinoma, through the regulation of stress homeostasis and tumor-microenvironment interactions [48]. Another study showed that SEL1L3 depletion enhances renal carcinoma cell growth and suppresses apoptosis by activating the ErbB/PI3K/mTOR signaling axis [53]. These phenotypic differences underscore the cell type-specific roles of SEL1L3 and the importance in various of cancers, but the underlining molecular mechanism remain elusive.

In the present study we identify HRD1 as a key binding partner of SEL1L3 and demonstrate that SEL1L3 impairs ERAD function via disrupting SEL1L-HRD1 complex stability and substrate recruitment. The study uncovers a novel mechanism of SEL1L3/HRD1/STING axis regulating colorectal cancer, potentially offering a new therapeutic target for its treatment.

## Materials and methods

### Cell lines and culture

The human colorectal cancer cell lines HT-29, HCT116, SW1116 and CACO2 were originally obtained from the American Type Culture Collection (ATCC). Human embryonic kidney cell line HEK-293T, SW1116 and CACO2 cells were cultured in Dulbecco’s modified Eagle’s medium (DMEM) (MA0212, Meilunbio) supplemented with 10% (v/v) FBS, and penicillin (50 U/ml)/streptomycin (50 μg/ml). HT-29 and HCT116 cells were maintained in Roswell Park Memorial Institute 1640 medium (RPMI 1640) containing 10% (v/v) FBS, penicillin (50 U/ml) and streptomycin (50 μg/ml). All cell lines were authenticated by DNA fingerprinting in the Shanghai Jiao Tong University Analysis Core and were cultured at 37 °C under 5% CO_2_ in a humidified chamber.

### Cell treatments

For ERAD treatments, the ubiquitination of HRD1 was inhibited using 1 μM LS-102 for 24 h (1456891-34-1, MedChemExpress) across all conditions. Cycloheximide (C1988, Sigma-Aldrich) was used at 10 μM. MG-132 (52801ES08, Yeasen Biotech) was used for 5 h at 10 μM concentration.

### Plasmids, transfection, and lenti-viral infection

PCR-based molecular cloning was employed to subclone various constructs into three distinct expression vectors: the inserts for Flag/HA-SEL1L3, Flag/HA/MYC-HRD1, HA-SEL1L, HA-OS9 and deletion mutants of HRD1 and SEL1L3 into the mammalian vector pCDNA3.1; those for 6His-HA-SEL1L3 and 6His-Flag-HRD1 into the prokaryotic vector pET-28a (Novagen); and those for HA/Flag-SEL1L3, HA/Flag-HRD1, HA-NHK and 3xFlag-STING into the viral vector pCDH-CMV-MCS-EF1-Puro (CD510B-1, System Biosciences), respectively. The pCDNA3.1-Flag/HA-HRD1-C1A variant was cloned from the pCDNA3.1-Flag/HA-HRD1 by point mutation of the first cysteine of the RING finger into an alanine. pGIPZ-shSEL1L3 plasmids were purchased from the DNA library of Shanghai Jiao Tong University School of Medicine (https://dnacore.shsmu.edu.cn/). The oligo of the short hairpin RNAs of human HRD1 and STING were inserted into pLKO.1-vector, to construct shHRD1 and shSTING plasmids. The short hairpin RNA sequences are listed in Supplementary Table S1.

Transient transfections were carried out using PEI (23966-2, Polysciences) or Lipofectamine 3000 reagent (L3000015, Invitrogen) according to the manufacturer’s instructions. Lentiviruses were harvested from HEK-293T cells and used to infect HEK-293T, HCT116, HT-29, SW1116 and CACO2 cells. Following infection, stable overexpression or knockdown cell lines were established by puromycin selection. For simultaneous overexpression or knockdown of SEL1L3 and HRD1, the corresponding lentiviruses were combined at a 1:1 ratio and used to infect the cells. The efficiency of overexpression and knockdown were assessed by quantitative real-time PCR (qPCR) and western blotting.

### Affinity purification of SEL1L3-interacting protein complex

To purify SEL1L3-associated proteins, pCDH-SEL1L3-Flag plasmids were stably expressed in HEK-293T cells. A total of 5 × 10^9^ cells were lysed in buffer A (20 mM Tris-HCl (pH 8.0), 150 mM NaCl, 2.5 mM EDTA, 0.5%NP-40, 0.2 mM PMSF, and 0.5 mM dithiothreitol (DTT)). For the immunoprecipitation we prepared cytoplasm extract from the cells. Although it is possible to perform immunoprecipitation on whole cell lysate, to minimize contaminating peptides and to maximize protein complex recovery, we routinely separate cytoplasm extract from nuclear components. A low-speed centrifugation step separates most of the cytosol from the intact nuclei. Cytoplasm extract was precleared with the protein A/G agarose (sc-2003, Santa Cruz) for 2 h and then incubated with the anti-Flag M2 affinity gels (F2426, Sigma-Aldrich) at 0.5 ml of beads per 100 mg of cell lysate for 2 h to overnight with rotation. The anti-Flag M2 gels were washed five times with buffer BC500 containing 20 mM Tris-HCl (pH 7.8), 500 mM KCl, 0.2 mM EDTA, 10% glycerol, 10 mM β-mercaptoethanol, 0.2% NP-40, 0.2 mM PMSF, and protease inhibitor cocktail. The protein complexes were eluted with the 3×Flag peptides (F4799, Sigma-Aldrich) at 0.4 mg/ml in buffer BC100 containing 20 mM Tris-HCl (pH 7.8), 50 mM KCl, 0.2 mM EDTA, 10% glycerol, 10 mM β-mercaptoethanol, 0.2 mM PMSF, and protease inhibitor cocktail. The eluted proteins were resolved on 4–12% SDS-PAGE gels for Western blotting and silver staining analyses. The specific bands were excised from the gels and identified by standard mass spectrometry.

### RNA extraction and quantitative real-time PCR (qRT-PCR)

Total RNA was extracted with TRIzol reagent (15596026, Invitrogen) following manufacturer’s instructions. RT-PCR was conducted on a Roche system (LightCycler 480II) using SYBR Green reagent (11188ES03, Yeasen) with the primers listed in Supplementary Table S1. All RT-PCR data were normalized against Actin and expressed as mean ± SD. Statistical differences between two groups were determined by unpaired two-tailed Student’s *t* test using Graphpad Prism (version 10.1.2). *p* ≤ 0.05 was considered statistically significant, and different levels of significance were expressed as follows: *P < 0.05; **P < 0.01; ***P < 0.001; ****P < 0.0001.

### Co-immunoprecipitation (co-IP), western blotting (WB), and antibodies

For co-IP assays, HEK-293T cells were transfected as indicated; 48 h later, cells were washed in ice-cold PBS, and then lysed in ice-cold IP lysis buffer (25 mM Tris-HCl (pH 7.4), 150 mM NaCl, 0.5% NP-40, 1 mM EDTA, 1 mM PMSF and protease inhibitor cocktail). After cell lysates were precleared with the protein A/G agarose for 2 h and then incubated with anti-Flag M2 affinity gels for 2 h at 4 °C. For reverse IP, HEK-293T cells were lysed and incubated with anti-HA affinity gels for 2 h at 4 °C. Agarose beads were washed three times with IP lysis buffer, boiled in SDS sample buffer, and then subjected to western blotting analysis. Western blotting was performed as previously described [54].

The antibodies were used for Western blotting as follows: rabbit anti-HRD1 (13473-1-AP), rabbit anti-SEL1L (29801-1-AP), mouse anti-α-GAPDH (60004-1-Ig), mouse anti-β-Actin (66009-1-Ig) and mouse anti-α-Tubulin (66031-1-Ig) were from Proteintech; rabbit anti-STING (13647), mouse anti-PDI(45596), rabbit anti-IgG (2729), and normal HRP-labeled secondary antibodies were from Cell Signaling Technology; rabbit anti-Flag (F7425), mouse anti-Flag (F1804), mouse anti-HA (H3663) and Flag M2 agarose beads (A2220) were from Sigma; anti-HA affinity gel (20586ES08) and mouse anti-ubiquitin (SC-807) were from Yeasen Biotech and Santa Cruz, respectively. The rabbit polyclonal SEL1L3 antibody was raised by immunizing rabbits with a chemically synthesized peptide of human SEL1L3 (QTIPPFERPFKDHQVC) as the antigen.

### Immunofluorescence (IF) and confocal microscopy

CACO2 cells stably expressing Flag-SEL1L3 were fixed in 4% Paraformaldehyde, permeabilized with 0.1% Triton X-100 in PBS, and blocked with 2% BSA. Cells were incubated with a cocktail of primary antibodies, including mouse anti-Flag (1:200), rabbit anti-Flag (1:200), rabbit anti-HRD1 (1:200) and mouse anti-PDI (1:200), diluted in blocking solution overnight at 4 °C. After three times washing, cells were then incubated with the secondary antibody mixture including 1:400 dilutions of Alexa Fluor 488 Donkey anti-Mouse IgG (A21202, Invitrogen) and Alexa Fluor 568 Donkey anti-Rabbit IgG (A10042, Invitrogen). Microscopy of the antibody- and DAPI-stained cells was performed with a Leica SP8 STED confocal microscope.

### Protein purification and size-exclusion fractionation assays

6his-HA-SEL1L3 and 6his-Flag-HRD1 proteins were expressed in E. coli. BL21 cells (CD601-02, TransGen). The proteins were extracted with binding buffer (50 mM Tris-HCl pH 7.4, 500 mM NaCl, 20 mM imidazole, 5 mM β-Mercaptoethanol, 1 mM PMSF, and protease inhibitor cocktail) under 4 °C. The slurry was lysed by High Pressure Cell Disruption, and centrifuged for 30 min at 20,000 rpm/min. The supernatant was loaded on a Ni-NTA column, and the protein was eluted with elution buffer (50 mM Tris-HCl pH 7.4, 500 mM NaCl, 500 mM imidazole, 5 mM β-Mercaptoethanol, 1 mM PMSF, and protease inhibitor cocktail). and concentrated with centrifugal filter devices (Amicon® Ultra-0.5 ml, 10 K) for immunoprecipitation and western blotting.

Flag-HRD1, HA-SEL1L3 and HA-SEL1L plasmids were transfected into HEK-293T cells for 48 h, cell lysates were incubated with anti-Flag M2 affinity gels for 4 h at 4 °C. The protein complexes were eluted with the 3 × Flag peptides at 0.4 mg/ml in buffer BC100. Fractionation of affinity eluate by HiLoad 16/600 Superdex 200 prep grade column (28989335, Cytiva) is carried out according to manufacturer’s instructions. Briefly, the column is equilibrated in buffer BC500, prior to loading 1–2 ml protein samples. We run the column at 2 ml/min and collect 2 ml fractions. All fractions were precipitated by Trichloroacetic Acid and resuspended in 50 μL 2 × SDS gel loading dye for western blotting.

### Cell proliferation and cell viability assays

Cell proliferation rate and cell viability were measured by cell counting kit-8 (MA0218, Meilunbio) and colony formation assay. For the CCK8 assay, Cells were seeded in 96-well plates at a density of 1 × 10^3^ cells per well in 200 μL complete medium for 0 d, 2 d, 4 d, 6 d and 8 d. After the cells were incubated with 10% CCK8 solution for 1 h, the absorbance at 450 nm was measured to determine the cell proliferation rate. For colony formation assay, 500 cells were seeded into each well of 12-well plate with 500 μL complete medium. After incubation at 37 °C for 10 days, the cell colonies were fixed with 4% paraformaldehyde, stained with 0.1% crystal violet, and then photographed.

### Cell migration and wound-healing assays

Migration of colorectal cancer cells was determined using 24-well Boyden chambers (CLS3422, Corning) with 8 μm-inserts. The detailed procedures of migration assay were previously described [54]. Exponentially growing HCT116, SW1116 or CACO2 cells were digested by trypsin and collected by centrifugation. The cells were resuspended in serum-free RPMI 1640 or DMEM blank medium and counted 3 × 10^4^ of HCT116, SW1116 or CACO2 cells in 100 μl serum-free RPMI 1640 or DMEM blank medium were seeded on 8 μm-inserts, then 600 μl RPMI 1640 or DMEM complete growth medium were added to the bottom chamber as attractants. After incubation for 48 h, the cells were fixed with 4% paraformaldehyde and stained with crystal violet, non-migrated cells on the top of the chamber were removed gently with cotton swabs, then migrated cells were counted as per field of view under phase contrast microscopy.

For the wound-healing assay, Cells (1 × 10^5^) were cultured in six-well plates. After incubating the seeded cells for 6 h at incubator with complete RPMI 1640 or DMEM medium, shaped wounds were scratched with a 10 μl pipette tip across each well after the cells reached over 90% confluence. The cells were gently washed with PBS twice to remove loose cells, and the complete medium was replaced with a low concentration of serum fresh medium (2%). To ensure that the wounds with the same wound area were comparable, multiple positioning marks were made at the center of the denuded surface. Then 0 h images were captured using a phase contrast microscope. Place the plates in incubator at 37 °C under 5% CO_2_ for 24 h, the second images were captured of the same wounds at 24 h. The scratch areas were measured and presented as the percentage of scratch areas at 0 h (mean ± SD). The percent of migration was calculated as the migration value in the scratch areas at 24 h divided by the scratch areas at 0 h. Each sample was performed at least three times.

### Tumor xenografting model

Male 7-week-old BALB/c nude mice were purchased from SLAC Laboratory Co. Ltd (Shanghai, China) and divided into four groups randomly. Stably transfected cells were injected into the flank of the mice (6 mice in each group). We measured the size of xenografts using calipers (calculated volume = shortest diameter^2^ *longest diameter *0.52) every 4 days. The subcutaneous tumors were harvested at 3 weeks and then measured and weighed. Blinding was implemented in this experiment. Animal studies were conducted following the Institutional Animal Care and Use Committee of Shanghai in accordance with the National Research Council Guide for Care and Use of Laboratory Animals (SCXK, Shanghai 2023-0010). All mice were fed under specific pathogen-free conditions. To ameliorate any suffering, mice were euthanized by CO_2_ inhalation.

### Statistical analysis

Please refer to the legend of the figures for description of sample sizes and statistical tests performed. Statistical analysis was performed using SPSS 27.0.1 (IBM Corp., Armonk, NY, USA) and Graph Pad Prism 10.1.2 (Graph Pad Software, Inc., La Jolla, CA, USA) software. The independent student’s *t* tests were used for comparison between two groups. The one-way ANOVA with Dunnett’s multiple comparisons test or two-way ANOVA with Sidak’s multiple comparisons test were used for comparison between three or more groups. Homogeneity of variances across groups was evaluated using Levene’s test or Bartlett’s test in this study. Differences were considered statistically significant when the *p* value was less than 0.05, and otherwise not significant (ns).

## Results

### SEL1L3 is a HRD1 interacting protein

**Fig. 1:**
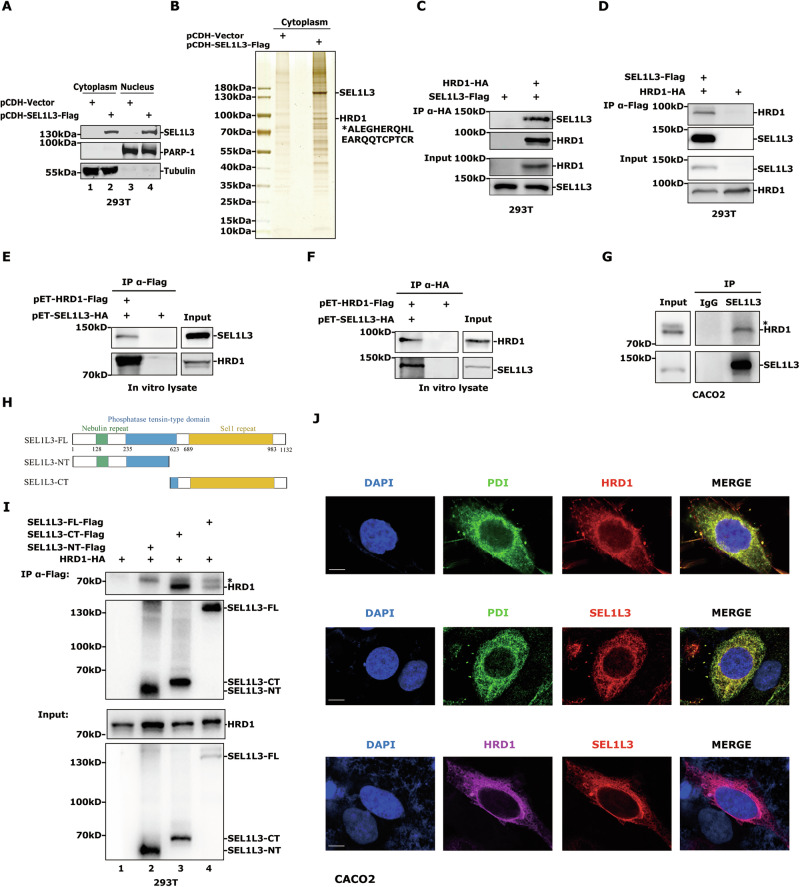
SEL1L3 interacts with HRD1 and co-localizes on the endoplasmic reticulum. **A** Western blot showed cytoplasmic, nuclear extracts and the protein expression of SEL1L3 in HEK-293T cells. **B** Silver staining showed that HRD1 was a potential SEL1L3 interacting protein. **C**, **D** Plasmids encoding Flag-SEL1L3 and HA-HRD1 were transiently transfected into HEK-293T cells, the cell lysates were incubated with either anti-HA beads or anti-Flag M2 beads and the co-eluted proteins were examined with anti-Flag (**C**) or anti-HA (**D**) antibodies. **E**, **F** In vitro interaction of SEL1L3 and HRD1. Bacterially expressed and purified full-length 6-his-HA-SEL1L3 and 6-his-Flag-HRD1 were used for in vitro binding assays. **G** Lysates prepared from CACO2 cells were incubated with anti-SEL1L3 antibody and protein A/G beads, and the co-eluted proteins were detected with an anti-HRD1 antibody. **H** Schematic diagram showed full-length and truncated SEL1L3 proteins. **I** HEK-293T cells were transfected with HA-HRD1 and Flag-SEL1L3 or its truncation mutants, the cell lysates were incubated with anti-Flag beads and immunoblotted for HA-HRD1. **J** Co-localization of SEL1L3 and HRD1 in CACO2 cells. Endogenous HRD1 and PDI in CACO2-Flag-SEL1L3 stable cells were detected for indirect immunofluorescence staining using anti-Flag, anti-HRD1 and anti-PDI antibodies, and the images were taken by confocal microscopy. Scale bar: 10 μm.

To elucidate the functional role of SEL1L3, we stably expressed Flag-SEL1L3 in HEK-293T cells and subjected to subcellular fractionation to separate nuclear and cytoplasmic components (Fig. 1A). Then, we performed affinity purification assays with cytoplasmic fraction of cell lysates with Flag beads. The co-eluents were resolved on SDS-PAGE followed by silver staining, and the specific band at 85 kDa was identified as HRD1 (Fig. 1B). To confirm the interaction between SEL1L3 and HRD1, we co-expressed HA-HRD1 and Flag-SEL1L3 in HEK-293T cells and performed reciprocal co-IP assays. Indeed, SEL1L3 and HRD1 interacted with each other (Fig. 1C, D). To determine if SEL1L3 directly binds HRD1, we employed full-length Flag-SEL1L3 and HA-HRD1 recombinant proteins prepared from *E. coli* for in vitro binding assays, and consistently Flag-SEL1L3 bound HA-HRD1 avidly (Fig. 1E, F). Moreover, a complex between endogenous SEL1L3 and HRD1 was readily detected in CACO2 cells (Fig. 1G).

To determine the regions in SEL1L3 responsible for HRD1 binding, SEL1L3 full-length or truncation mutants were transiently co-expressed with HA-HRD1 in HEK-293T cells and co-IP assays demonstrated that C-terminal domain of SEL1L3 protein displayed strong binding activity toward HRD1, which was much more than that of full-length SEL1L3, whereas the N-terminal SEL1L3 showed no binding activity (Fig. 1H, I). Moreover, to determine the subcellular localization of SEL1L3 and HRD1, indirect immunofluorescence assays were performed in CACO2 cells using antibodies specifically against SEL1L3 or HRD1, with PDI as an ER marker (Fig. 1J). Notably, both SEL1L3 and HRD1 proteins co-localized in the endoplasmic reticulum. Collectively, these data demonstrate that SEL1L3 is a HRD1 binding partner both in vitro and in vivo.

### SEL1L3 impairs HRD1-mediated ERAD activity

**Fig. 2:**
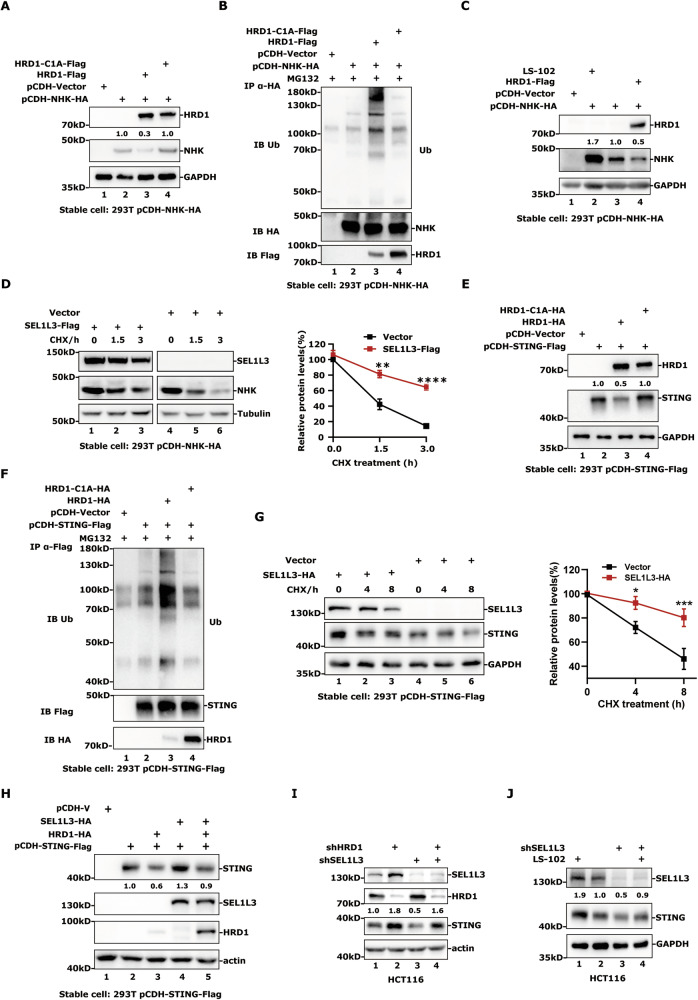
SEL1L3 inhibits the degradation of HRD1 substrates NHK and STING. **A** Western blot analysis of NHK protein levels was performed in HEK-293T cells stably expressing HA-NHK and transiently transfected with Flag-HRD1 or Flag-HRD1-C1A. **B** HEK-293T cells stably expressing HA-NHK were transfected with Flag-HRD1 or Flag-HRD1-C1A followed by treatment with MG-132. HA-NHK was immunoprecipitated and immunoblotted for total ubiquitination. **C** Western blot analysis of NHK protein levels in HEK-293T cells stably expressing HA-NHK transfected with Flag-HRD1 or treated with LS-102. **D** Western blot analysis of NHK protein levels in CHX-treated HEK-293T cells stably expressing HA-NHK transfected with vector or Flag-SEL1L3 (left panel), and quantification of protein decay (right panel). **E** Western blot analysis of STING protein levels in HEK-293T cells stably expressing Flag-STING and transiently transfected with HA-HRD1 or HA-HRD1-C1A. **F** HEK-293T cells stably expressing Flag-STING were transfected with HA-HRD1 or HA-HRD1-C1A followed by treatment with MG-132. Flag-STING was immunoprecipitated and immunoblotted for total ubiquitination. **G** Western blot analysis of STING protein levels in CHX-treated HEK-293T cells stably expressing Flag-STING transfected with vector or HA-SEL1L3 (left panel), and quantification of protein decay (right panel). **H** Western blot analysis of STING protein levels in HEK-293T cells stably expressing Flag-STING transfected with HA-HRD1 and HA-SEL1L3 either individually or together. **I** Western blot analysis of STING protein levels in HCT116 cells with SEL1L3 and HRD1 depletion either individually or together. **J** Western blot analysis of STING protein levels in HCT116-shVector and HCT116-shSEL1L3 cells treated with or without LS-102. Three independent experiments were performed, data were shown as mean ± SD, ****P < 0.0001, ***P < 0.001, **P < 0.01, *P < 0.05, Student *t* test. CHX cycloheximide. NHK a mutation variant of Alpha-1 Antitrypsin.

To examine the effect of SEL1L3 on HRD1-mediated degradation, we stably expressed NHK mutant in HEK-293T cells, a variant of Alpha-1 Antitrypsin and a known substrate of HRD1 complex [46]. Consistent with previous reports, expression of normal HRD1 apparently decreased NHK protein levels (Fig. 2A), and concomitantly, HRD1 prompted NHK polyubiquitylation in HEK-293T cells (Fig. 2B). However, the HRD1 mutant (HRD1 C1A), which lacks the E3 ligase activity, had little to no effect on NHK protein levels and ubiquitination (Fig. 2A, B). LS-102, a specific inhibitor of HRD1 [55] caused a dramatic increase in NHK protein levels (Fig. 2C). Similarly, expression of SEL1L3 maintained NHK stability and reduced its degradation rate, which demonstrate that SEL1L3 inhibits HRD1‑mediated degradation of NHK (Fig. 2D).

Emerging evidence has demonstrated that the HRD1-ERAD machinery can degrade STING [33]. Similar to NHK protein, expression of normal HRD1, but not mutant HRD1 (HRD1 C1A), apparently decreased STING protein levels (Fig. 2E), and robustly ubiquitinated STING protein in HEK-293T-Flag-STING cells (Fig. 2F). Conversely, expression of SEL1L3 increased STING protein levels and reduced the degrading rate of STING protein (Fig. 2G). Next, we co-expressed HA-HRD1 and HA-SEL1L3 either individually or together in HEK-293T-Flag-STING cells. Western blot assays showed that expression of HRD1 alone markedly reduced STING protein levels, while SEL1L3 alone increased STING protein. Notably, co-expression of SEL1L3 and HRD1 overrode the effect of HRD1 on STING protein (Fig. 2H). To assess the effect of SEL1L3 on endogenous STING protein levels, stable expression of shRNAs specifically targeting SEL1L3 in HCT116 cells apparently decreased STING protein levels, which can be reversed by the HRD1 inhibitor LS-102 or depletion of protein HRD1 (Fig. 2I, J). Together, these data suggest that SEL1L3 can impair HRD1-mediated ubiquitination and degradation of its substrates.

### SEL1L3 impairs ERAD substrate recruitment through disruption of the SEL1L-HRD1 complex

**Fig. 3:**
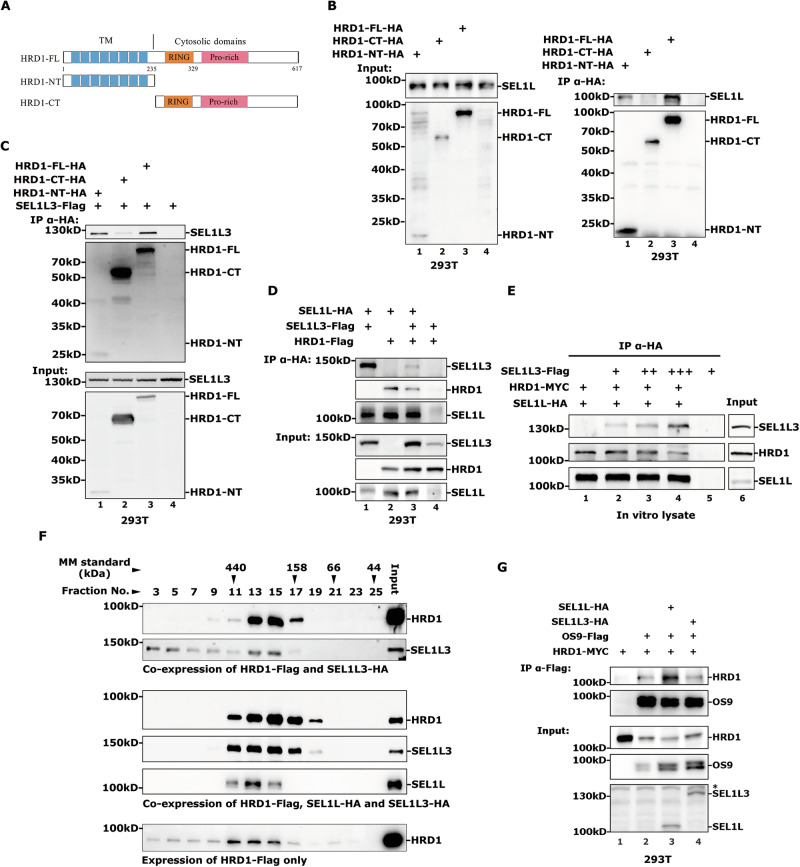
SEL1L3 impairs ERAD substrate recruitment through disruption of the SEL1L-HRD1 complex. **A** Schematic diagram showed full-length and truncated HRD1 proteins. **B** HEK-293T cells were transfected with HA-HRD1 or its truncation mutants, the cell lysates were incubated with anti-HA beads and immunoblotted for SEL1L. **C** HEK-293T cells were transfected with Flag-SEL1L3 and HA-HRD1 or its truncation mutants, the cell lysates were incubated with anti-HA beads and immunoblotted for Flag-SEL1L3. **D** HEK-293T cells were transfected with HA-SEL1L, Flag-HRD1 and Flag-SEL1L3. HA-SEL1L was immunoprecipitated from cell lysates and immunoblotted for Flag-HRD1 and Flag-SEL1L3. **E** In vitro interaction of SEL1L3, HRD1 and SEL1L. Lysates prepared from HEK-293T cells transfected with Flag-SEL1L3, MYC-HRD1 and HA-SEL1L, respectively, were used for in vitro binding assays. **F** Western blot showed the patterns of the HRD1, SEL1L3 or SEL1L proteins eluted from HiLoad 16/600 Superdex 200 prep grade column. Whole-cell extract (8 mg) was prepared from HEK-293T cells overexpressing Flag-SEL1L3, Flag-HRD1 and HA-SEL1L, the cell lysates were incubated with anti-Flag beads and the co-eluted proteins were loaded onto a HiLoad 16/600 Superdex 200 prep grade column. **G** HEK-293T cells were transfected with Flag-OS9, MYC-HRD1 and HA-SEL1L3 or HA-SEL1L. Flag-OS9 was immunoprecipitated from cell lysates and immunoblotted for MYC-HRD1. *non-specific band.

SEL1L plays an essential role in the ERAD process via facilitating the recognition and dislocation of misfolded proteins to HRD1 and concomitantly increases the stability of HRD1 [26, 44]. Consistent with the previous report, we showed that SEL1L bound the N-terminal region of the HRD1 protein. (Fig. 3A, B). Next, we carried out co-IP assays to examine if SEL1L3 also binds the N-terminal region of the HRD1. HRD1 full-length or truncation mutants were transiently co-expressed with Flag-SEL1L3 in HEK-293T cells and co-IP assays demonstrated that N-terminal domain of HRD1 protein displayed strong binding activity toward SEL1L3, which was comparable to that of full-length HRD1, whereas the HRD1 protein C-terminus showed weak or no binding activity (Fig. 3C).

To determine the effect of SEL1L3 on HRD1-SEL1L interaction, we transfected plasmids encoding HRD1, SEL1L3 and SEL1L alone or in combinations in HEK-293T cells, and co-IP assays were performed subsequently with anti-HA beads. Notably, SEL1L could readily immunoprecipitate SEL1L3 and HRD1, respectively, whereas co-expression of SEL1L, SEL1L3, and HRD1 resulted in lesser immunopreciated proteins of HRD1 and SEL1L3 (Fig. 3D). Moreover, increasing the amount of SEL1L3 protein progressively decreased HRD1-SEL1L interactions (Fig. 3E), suggesting a direct mutual binding competition exists among SEL1L, SEL1L3, and HRD1 proteins. To further strengthen this observation that SEL1L3 is not a component of the HRD1-SEL1L complex, we performed size-exclusion fractionation assays with whole-cell extracts prepared from HEK-293T cells expressing Flag-HRD1, HA-SEL1L3, or HA-SEL1L. When HRD1 and SEL1L3 were co-expressed, both proteins were eluted and peaked in fractions 13-15; co-expression of SEL1L, SEL1L3 and HRD1 resulted in apparent pattern changes: the HRD1 and SEL1L3 proteins were peaked in fractions 13–17, while SEL1L was eluted in a single peak of fraction 13 (Fig. 3F). In contrast, expression of HRD1 alone, the protein was eluted in fractions 11-15 (Fig. 3F, bottom panel).

Literatures have reported that SEL1L is required for the formation of an HRD1 functional complex by recruiting ER chaperones like OS9 to HRD1 [45, 46]. To investigate whether SEL1L3-mediated disruption of SEL1L-HRD1 complex compromises substrate recruitment, we co-expressed HA-SEL1L3 or HA-SEL1L, MYC-HRD1 and Flag-OS9 in HEK-293T cells. Co-IP assays demonstrated that SEL1L facilitated the binding between OS9 and HRD1, whereas SEL1L3 showed no such effect (Fig. 3G). Together, these data suggest that SEL1L3 disrupts the binding between HRD1 and SEL1L.

### SEL1L3 promotes the degradation of HRD1 protein

**Fig. 4:**
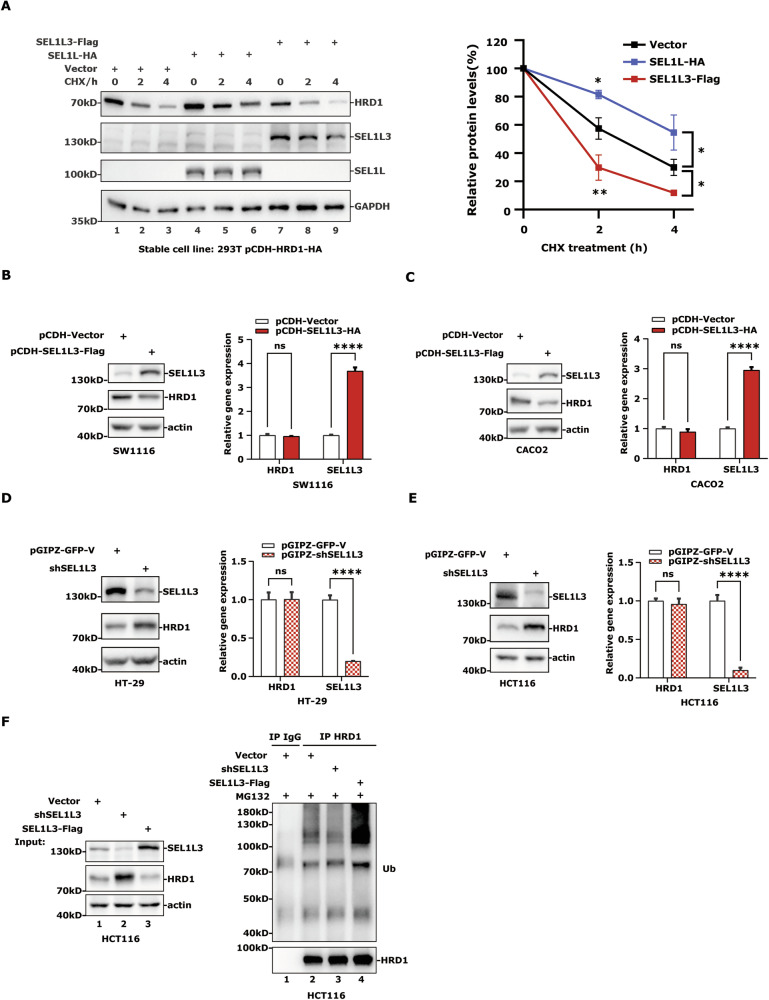
SEL1L3 promotes HRD1 degradation. **A** Western blot analysis of HRD1 protein levels in CHX-treated HEK-293T cells stably expressing HA-HRD1 transfected with vector, Flag-SEL1L3 or HA-SEL1L (left panel), and quantification of protein decay (right panel). Data were shown as mean ± SD, **P < 0.01, *P < 0.05, Ordinary one-way ANOVA with Dunnett’s multiple comparisons test. **B**, **C** Western blot analysis of HRD1 protein levels in SW1116 cells (**B**) and CACO2 cells (**C**) with SEL1L3 overexpression (left panel), and RT-PCR showed the mRNA levels of HRD1 in CRC cells with stably expressing SEL1L3 (right panel). **D**, **E** Western blot analysis of HRD1 protein levels in HT-29 cells (**D**) and HCT116 cells (**E**) with SEL1L3 knockdown, and RT-PCR showed the mRNA levels of HRD1 in CRC cells with SEL1L3 knockdown (right panel). **F** HCT116 vector cells, HCT116 cells stably expressing Flag-SEL1L3 and HCT116 SEL1L3-knockdown cells were treated with MG-132. HRD1 was immunoprecipitated from cell lysates and immunoblotted for total ubiquitination. Three independent experiments were performed, data were shown as mean ± SD, ****P < 0.0001, ns: no significance, Student *t* test.

To determine whether SEL1L3 can affect HRD1 protein stability, we stably expressed HRD1 in HEK-293T cells, plasmids encoding Flag-SEL1L3 and HA-SEL1L were transfected into HEK-293T-HA-HRD1 cells individually. The result showed that expression of SEL1L markedly increased HRD1 protein levels and reduced its degradation rate as previously reported, while SEL1L3 progressively decreased HRD1 protein levels without change its mRNA levels (Fig. 4A, Fig. S1A). Furthermore, expression of SEL1L3 in SW1116 and CACO2 cells resulted in decreased endogenous HRD1 protein levels (Fig. 4B, C). Conversely, stable expression of shRNAs specifically targeting SEL1L3 in HT-29 or HCT116 cells apparently increased HRD1 protein levels (Fig. 4D, E). Further, expression of SEL1L3 markedly increased ubiquitination of HRD1, in contrast depletion of SEL1L3 resulted in decreased HRD1 ubiquitination levels (Fig. 4F). Collectively, these data demonstrate that SEL1L3 can accelerate HRD1 degradation by increasing its ubiquitination.

### SEL1L3 antagonizes HRD1-mediated CRC cell growth and migration

**Fig. 5:**
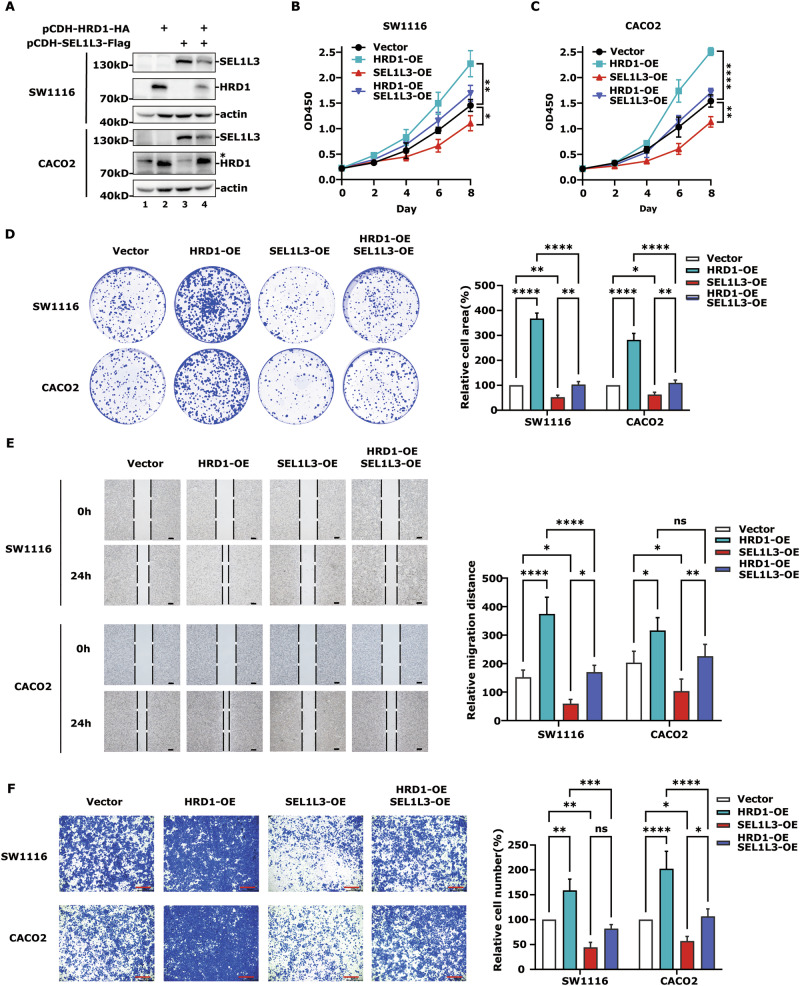
SEL1L3 suppresses CRC cell growth and migration through functional suppression of HRD1. **A** Western blot analysis of SEL1L3 and HRD1 protein levels. Overexpression of SEL1L3 and HRD1 in SW1116 and CACO2 cells, respectively. *non-specific band. **B, C** CCK8 assays were performed in SW1116 (**B**) and CACO2 (**C**) cells with simultaneous or individual overexpression of SEL1L3 and HRD1. **D, E, F** Colony formation (**D**), wound healing (**E**) and transwell assays (**F**) were performed in SW1116 and CACO2 cells with simultaneous or individual overexpression of SEL1L3 and HRD1. Scale bar: 100 μm. Data were shown as mean ± SD of four independent experiments. ****P < 0.0001, ***P < 0.001, **P < 0.01, *P < 0.05, ns no significance, two-way ANOVA with Sidak’s multiple comparisons test.

To investigate the biological function of SEL1L3 in CRC, we stably expressed SEL1L3, HRD1 alone or in combination in SW1116 and CACO2 cells, and their expression levels were examined by western blot assays (Fig. 5A, Fig. S1B). CCK8 and colony formation assays demonstrated that expression of HRD1 accelerated cell growth and colony numbers, while expression of SEL1L3 inhibited cell growth and reduced colony numbers; notably, simultaneous expression of HRD1 and SEL1L3 offset their effect on cell growth and colony formation (Fig. 5B, C, D). Similarly, expression of SEL1L3 inhibited the migration ability, while expression of HRD1 accelerated cell migration of SW1116 and CACO2 cells; co-expression of SEL1L3 and HRD1 reciprocally offset their effect on cell migration (Fig. 5E, F).

### Depletion of SEL1L3 accelerates the proliferation and migration of CRC cells

**Fig. 6:**
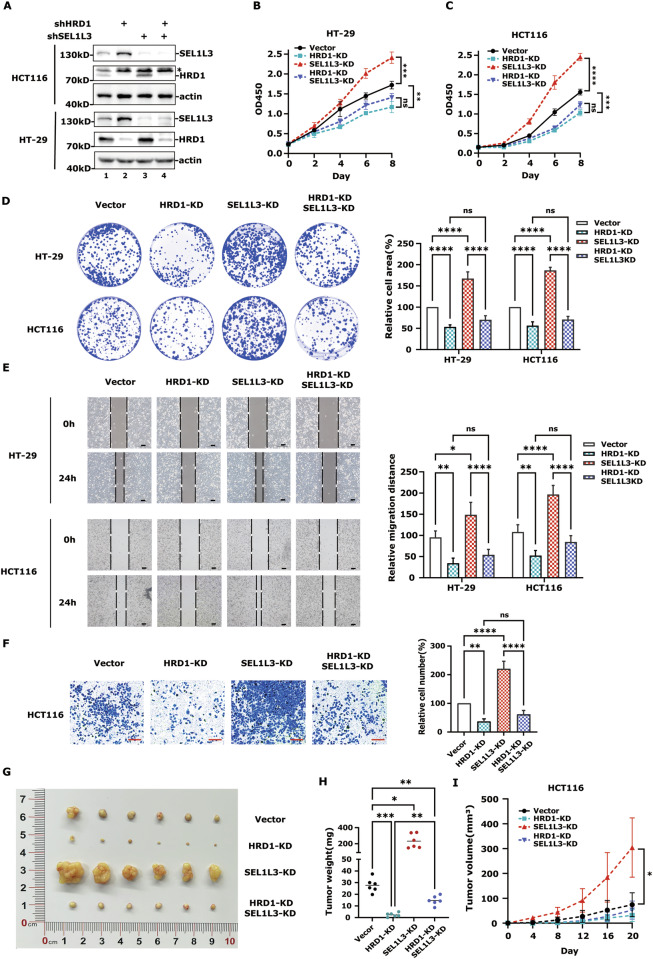
Depletion of SEL1L3 promotes tumor growth and is reversed by depletion of HRD1 in CRC cells. **A** Western blot analysis of SEL1L3 and HRD1 protein levels. Knocking-down of SEL1L3 and HRD1 in HT-29 and HCT116 cells, respectively. *non-specific band. **B, C** CCK8 assays were performed in HT-29 (**B**) and HCT116 (**C**) cells with simultaneous or individual knockdown of SEL1L3 and HRD1. Data were shown as mean ± SD of four independent experiments. **D, E** Colony formation (**D**) and wound healing assays (**E**) were performed in HT-29 and HCT116 cells with simultaneous or individual knockdown of SEL1L3 and HRD1. Scale bar: 100 μm. Data were shown as mean ± SD of four independent experiments. **F** Transwell assay was performed in HCT116 cells with simultaneous or individual knockdown of SEL1L3 and HRD1. Scale bar: 100 μm. Data were shown as mean ± SD of four independent experiments. **G** Images of xenografts in male BALB/c nude mice (*n* = 6). **H** Tumor weight was measured after 3 weeks. **I** Tumor volume was measured every four days in different groups. Data were shown as mean ± SD. ****P < 0.0001, ***P < 0.001, **P < 0.01, *P < 0.05, ns: no significance, two-way ANOVA with Sidak’s multiple comparisons test.

To further interrogate the antagonizing role of SEL1L3 against HRD1, we depleted HRD1 and/or SEL1L3 proteins by stable expression of shRNAs specifically targeting SEL1L3 or HRD1 genes in HT-29 and HCT116 cells, protein levels were confirmed by western blot assays (Fig. 6A). Consistently, depletion of SEL1L3 accelerated cell growth, colony formations, and cell migration, while depletion of HRD1 resulted in decreased cell growth, colony formations and cell migration compared to vector control cells (Fig. 6B, C, D, E, F). Surprisingly, the pro-tumor effect induced by SEL1L3 knockdown markedly decreased when HRD1 protein was concomitantly depleted, demonstrating that tumor suppression function of SEL1L3 requires the presence of HRD1 (Fig. 6B, C, D, E, F).

To verify the role of SEL1L3 and HRD1 on tumor growth in vivo, HCT116 cells bearing shSEL1L3, shHRD1 alone or in combination, or mock vector were subcutaneously injected into 7-week-old BALB/c nude mice. Three weeks after post-inoculation, xenografted tumors were all successfully established and tumor growth was measured every 4 days. Similar to the observations in cell assays, depletion of SEL1L3 markedly promoted tumor growth, while depletion HRD1 significantly suppressed tumor growth compared to that of the controls; tumors formed by cells bearing double depletion of SEL1L3 and HRD1 showed a significantly lower weight compared to the vector group, but a higher weight than those with HRD1 knockdown alone (Fig. 6G, H). In addition, the growth rate was similar to tumor weight (Fig. 6I). Collectively, these data indicate that SEL1L3 exerts a tumor-suppressing function by antagonizing HRD1 function.

### STING is required for the tumor suppression function of SEL1L3

**Fig. 7:**
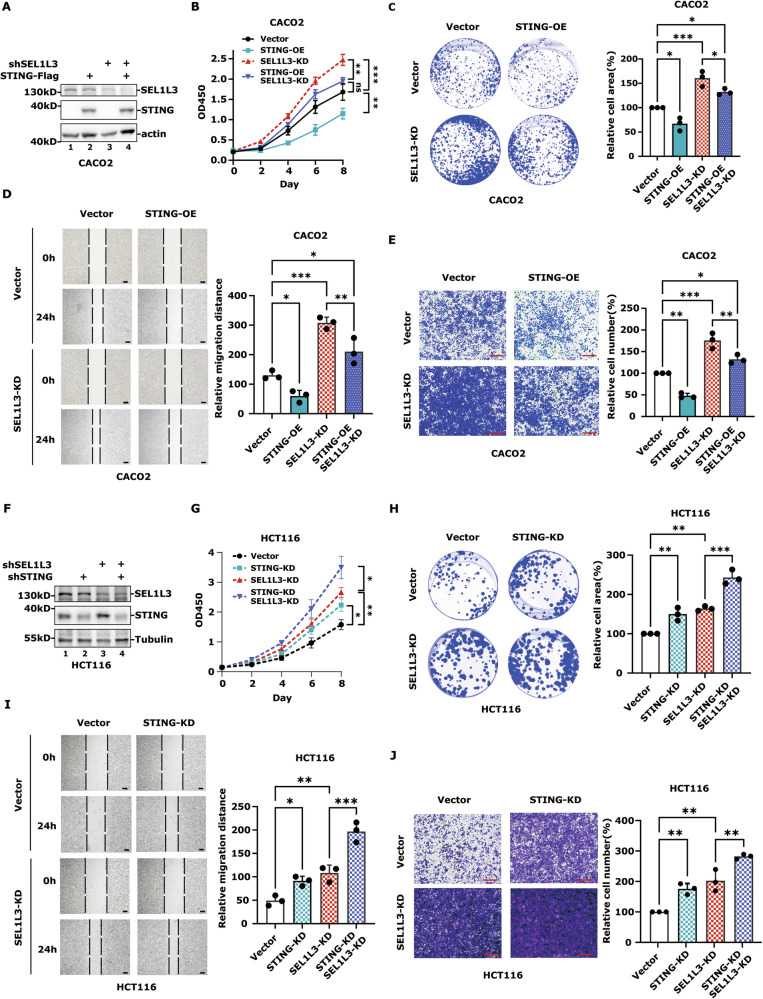
STING is an important downstream molecule mediating the tumor suppression function of SEL1L3. **A** Western blot analysis of SEL1L3 and STING protein levels. Knocking-down of SEL1L3 in STING overexpression CACO2 cells. **B, C, D, E** CCK8 (**B**), colony formation (**C**), wound healing (**D**), and transwell assays (**E**) were performed in CACO2-shSEL1L3 cells with STING rescue. **F** Western blot analysis of SEL1L3 and STING protein levels. Knocking-down of SEL1L3 in STING-depletion HCT116 cells. **G, H, I, J** CCK8 (**G**), colony formation (**H**), wound healing (**I**), and transwell assays (**J**) were performed in HCT116-shSEL1L3 cells with STING knockdown. Scale bar: 100 μm. Data were shown as mean ± SD of three independent experiments. ***P < 0.001, **P < 0.01, *P < 0.05, ns: no significance, two-way ANOVA with Sidak’s multiple comparisons test.

Since SEL1L3 can stabilize STING protein through inhibiting the ubiquitination function of HRD1 (Fig. 2H, I, J), we speculated that SEL1L3 may exert its biological functions by modulating the STING signaling pathway in CRC cells. Thus, we depleted SEL1L3 in CACO2-Vector and CACO2-STING cells (Fig. 7A), CCK8 and colony formation assays showed depletion of SEL1L3 in vector cells markedly accelerated cell growth and colony numbers, while depletion of SEL1L3 in CACO2-STING cells showed less growth rate and colony numbers (Fig. 7B, C). Similarly, overexpression of STING dampened the acceleration effect on cell migration of SEL1L3 (Fig. 7D, E).

To strengthen the evidence, we stably expressed shSEL1L3 and shSTING, either individually or together in HCT116 cells (Fig. 7F). Consistently, SEL1L3 depletion accelerated cell proliferation and migration, while STING depletion overrode effects of depletion of SEL1L3 mediated cell proliferation and migration (Fig. 7G, H, I, J). Together, these data demonstrate that STING is an important downstream molecule mediating the tumor suppression function of SEL1L3.

**Fig. 8:**
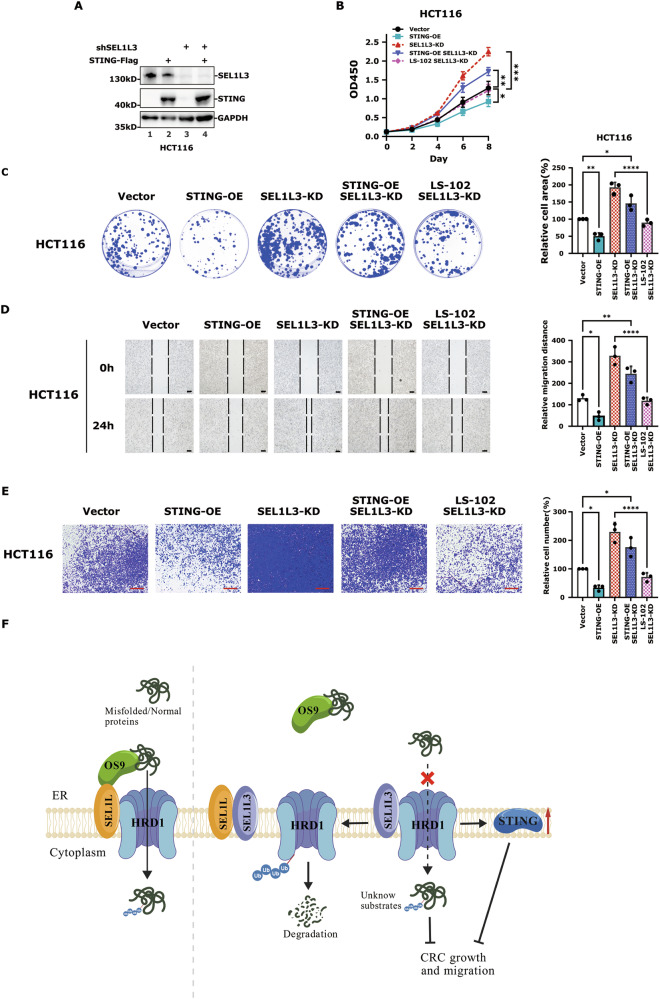
The SEL1L3/HRD1/STING axis plays a crucial role in CRC growth and metastasis. **A** Western blot analysis of SEL1L3 and STING protein levels. Knocking-down of SEL1L3 in STING overexpression HCT116 cells. **B, C, D, E** CCK8 (**B**), colony formation (**C**), wound healing (**D**), and transwell assays (**E**) were performed in HCT116-shSEL1L3 cells with STING rescue. Scale bar: 100 μm. Data were shown as mean ± SD of three independent experiments. ****P < 0.0001, ***P < 0.001, **P < 0.01, *P < 0.05, ns: no significance, two-way ANOVA with Sidak’s multiple comparisons test. **F** Schematic of the mechanism of SEL1L3/HRD1/STING axis in modulating CRC growth and metastasis.

To determine whether the SEL1L3-STING axis inhibits CRC cell growth and migration through HRD1, we depleted SEL1L3 in HCT116-Vector and HCT116-STING cells (Fig. 8A), and a HRD1 inhibitor LS-102 was added in stably SEL1L3 depletion HCT116 cells. Consistently, CCK8 and colony formation assays showed depletion of SEL1L3 in vector cells markedly accelerated cell growth and colony numbers, while STING overexpression or HRD1 inhibitor LS-102 could reverse the status, which showed less growth rate and colony numbers (Fig. 8B, C). Similarly, overexpression of STING or HRD1 inhibitor LS-102 dampened the acceleration effect on cell migration of SEL1L3 (Fig. 8D, E). These observations demonstrate that the SEL1L3/HRD1/STING axis plays a crucial role in CRC growth and metastasis.

## Discussion

ERAD is a crucial quality and quantity control system of protein synthesis, which must be tightly adjusted in accordance with specific needs of cells, however, the factors that govern ERAD activity remain largely unidentified. Here in this paper, we demonstrate that SEL1L3 can impede substrate degradation activity of ERAD via dual mechanisms: SEL1L3 disrupts SEL1L-HRD1 complex and concomitant prevention of substrate recruitment by mutually exclusively binding HRD1 and SEL1L; on the other hand, SEL1L3 can accelerate HRD1 protein degradation. Biologically, SEL1L3 exerts a tumor-suppressing function evidenced by inhibiting CRC cell growth and migration; moreover, we identify STING as an HRD1 substrate and a critical downstream molecule mediating tumor suppression activity of SEL1L3 (Fig. 8F). These results unveil a potential regulatory molecule for ERAD, applicable not only in cancer cells but also in normal cells.

Among ERAD pathways, the SEL1L-HRD1 complex is the most evolutionarily conserved from yeast to mammals [11, 24]. HRD1 forms the retro-translocation channel [14, 15], where SEL1L acts as an adaptor to ensure HRD1 stability and substrate recognition [25, 26, 44]. Deletion of SEL1L or HRD1 results in embryonic or early postnatal lethality, indicating their essential roles in development and tissue homeostasis [40, 41]. Moreover, genetic ablation studies with cellular specificity have elucidated the critical contributions of the SEL1L-HRD1 ERAD in diverse physiological processes, encompassing lipid and glucose metabolism, immunomodulation, thermogenesis, and organ homeostasis [3, 11, 36]. SEL1L3 protein belongs to the SEL1L family, but its specific role within the cell remains unclear.

Our study showed that SEL1L3 interacts with HRD1, and impedes substrate ubiquitination activity of HRD1. Co-IP and Gel Filtration assays showed that SEL1L3, HRD1 and SEL1L interact in a pairwise manner, but not co-exist in one ternary complex, indicating these three proteins display mutually exclusive interaction patterns. Moreover, SEL1L3 plays essential roles in HRD1 protein stability and substrate recruitment by disrupting the HRD1-SEL1L interaction. Collectively, these findings not only demonstrate the mechanism for SEL1L3-mediated inhibition of HRD1, but also offer novel insights into the regulation of ERAD process.

SEL1L3 plays a vital role in ERAD, but its biological function is not defined. Recent studies have also highlighted the role of ERAD in the development of CRC. CRC cells exhibit a higher level of ER stress relative to adjacent non-tumor tissues [56], and previous research reported that HRD1 facilitates migration and invasion in CRC by promoting the expression of MMP-2 and MMP-9 [57], indicating that targeting ER stress-related signaling may offer a novel strategy to overcome CRC. In this study, we first showed that the SEL1L3 downregulation promotes the tumorigenesis, proliferation, invasion, and metastasis of CRC, and can antagonize the oncogenic role of HRD1.

Activation of cGAS-STING pathway in CRC triggers the production and release of IFN-I and multiple inflammatory cytokines, thereby initiating an anti-tumor immune response which in turn suppresses CRC progression [58]. However, limited research has focused on the impact of the STING protein on the development and progression of CRC. In our study, we demonstrated that STING is a critical downstream molecule of SEL1L3 to mediate its tumor-suppression function. Either depletion or expression of STING in CRC can markedly dampen or enhance SEL1L3-mediated cell growth and migration, although other molecules may exist to mediate the tumor-suppression function of SEL1L3. These observations demonstrate that the SEL1L3/HRD1/STING axis plays a crucial role in CRC growth and metastasis.

In summary, our study demonstrates that SEL1L3 is a crucial regulator of HRD1-mediated ERAD, and exerts a tumor-suppression function in CRC. Although we have confirmed that SEL1L3 inhibits HRD1-mediated ubiquitination function and stabilizes its substrate proteins, such as NHK and STING, it remains to be determined which substrate affected by SEL1L3 among the numerous HRD1 substrates can also participate in tumor suppression. Moreover, the mechanisms by which SEL1L3 competes with SEL1L for HRD1 binding and the identification of additional critical components within the SEL1L3-HRD1 complex merit further exploration.

## Supplementary information

Supplementary legends

Supplementary Figure S1

Original Data

Supplementary Table S1

## Data Availability

The data, protocols and sequences that support the findings of this study are available from the corresponding authors upon reasonable request.
