# Supplementary material for: SEL1L3 suppresses colorectal cancer cell growth and metastasis by preventing endoplasmic reticulum-associated degradation of STING
Source: Cell Death Dis. 2026 May 3;17(1):586. doi: 10.1038/s41419-026-08770-6 (PMC13280011; doi:10.1038/s41419-026-08770-6)
Supplement: Supplementary file 1 — Supplementary legends [file 41419_2026_8770_MOESM1_ESM.docx]

**Fig. S1 A.** RT-PCR showed the mRNA levels of HRD1 in stably overexpression HRD1 HEK-293T cells with transiently expressing SEL1L3 or SEL1L. ns: no significance, Ordinary one-way ANOVA and Dunnett’s multiple comparisons tests. **B.** Western blot analysis of SEL1L3 and HRD1 protein levels in colorectal cancer cells. *non-specific band.
