# Supplementary figures and images for: SEL1L3 suppresses colorectal cancer cell growth and metastasis by preventing endoplasmic reticulum-associated degradation of STING
Source: Cell Death Dis. 2026 May 3;17(1):586. doi: 10.1038/s41419-026-08770-6 (PMC13280011; doi:10.1038/s41419-026-08770-6)

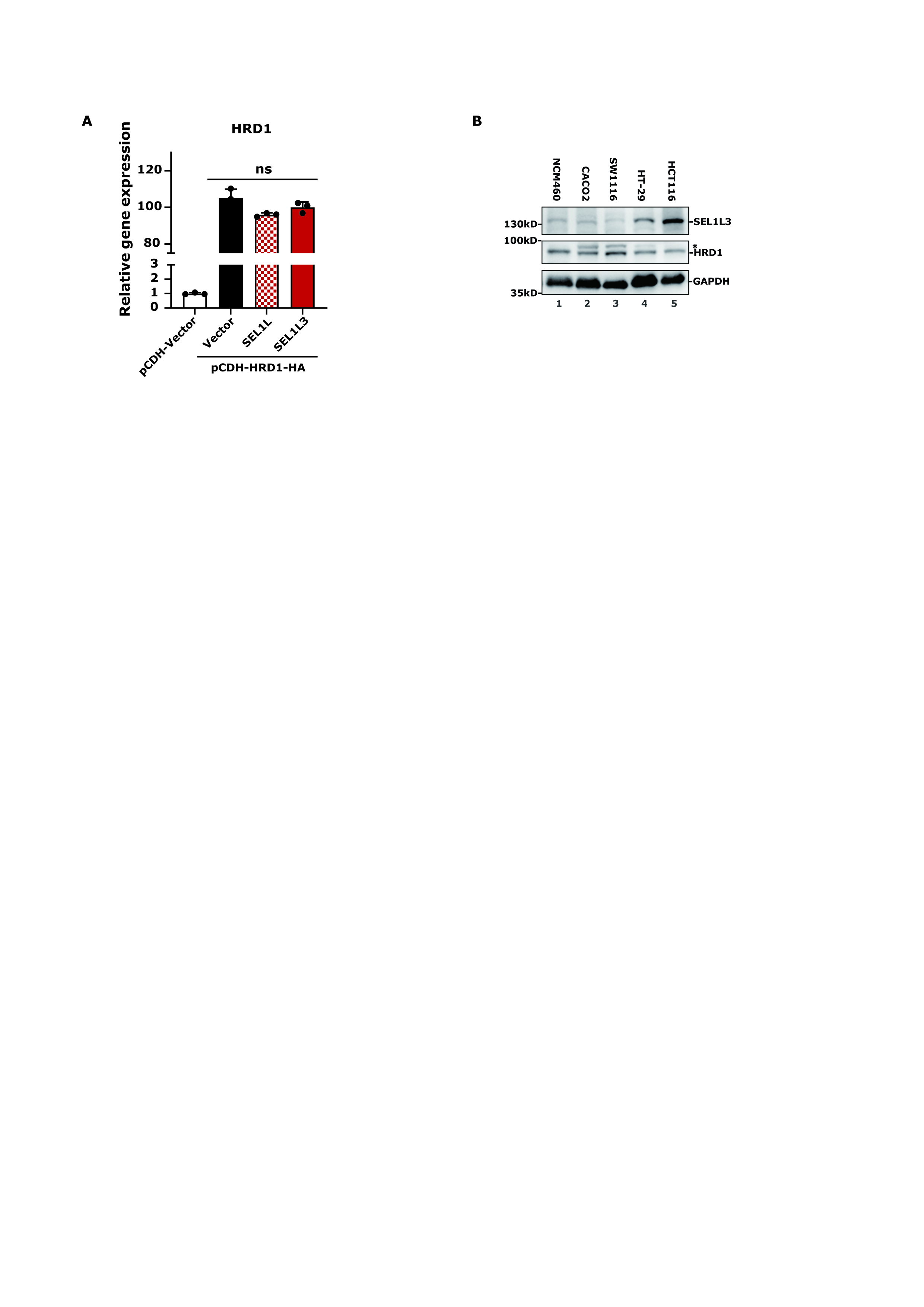

Supplement: Supplementary file 2 — Supplementary Figure S1 [file 41419_2026_8770_MOESM2_ESM.tif]

**Figure 1**

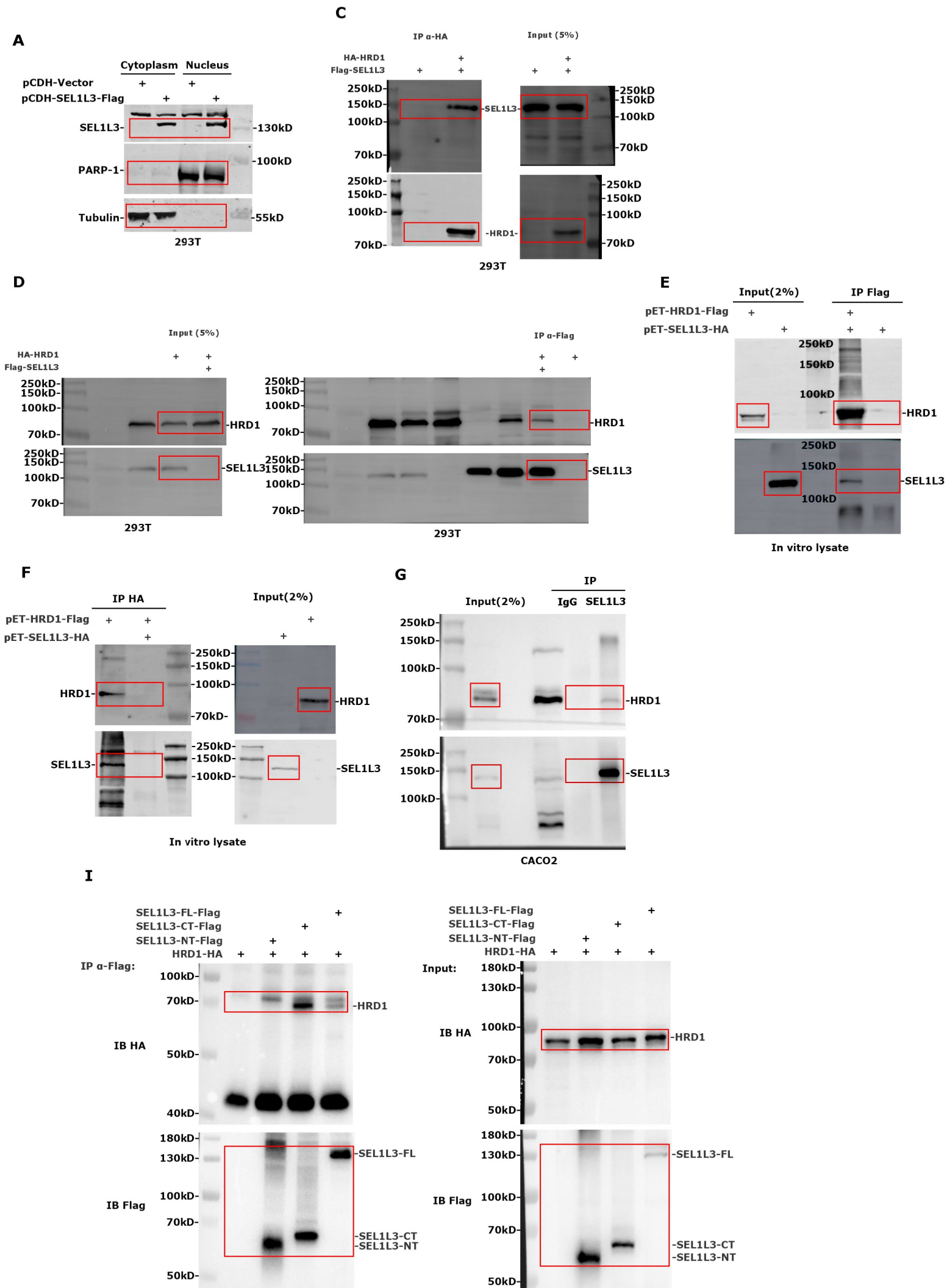

**Figure 2**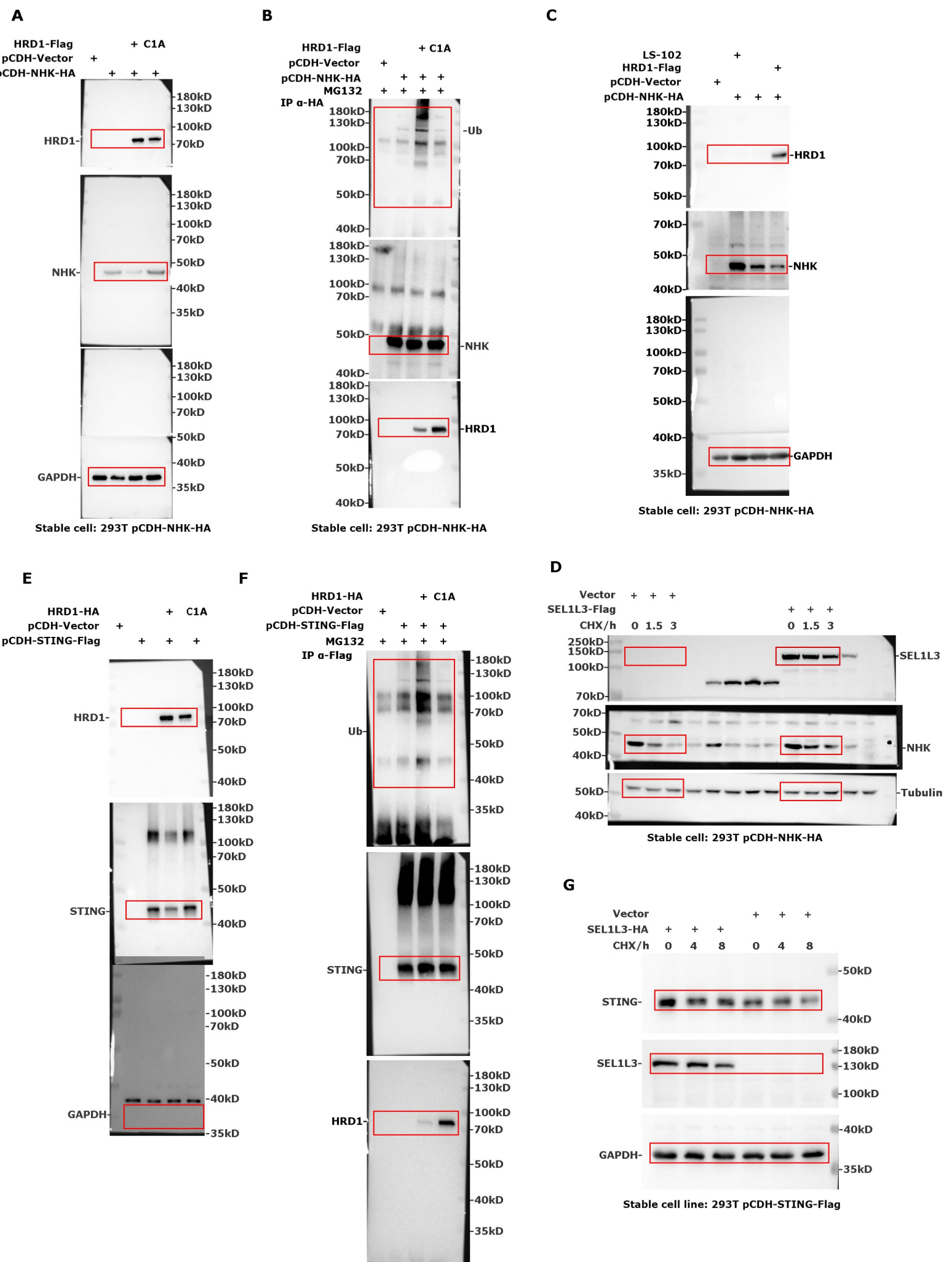

Figure 2

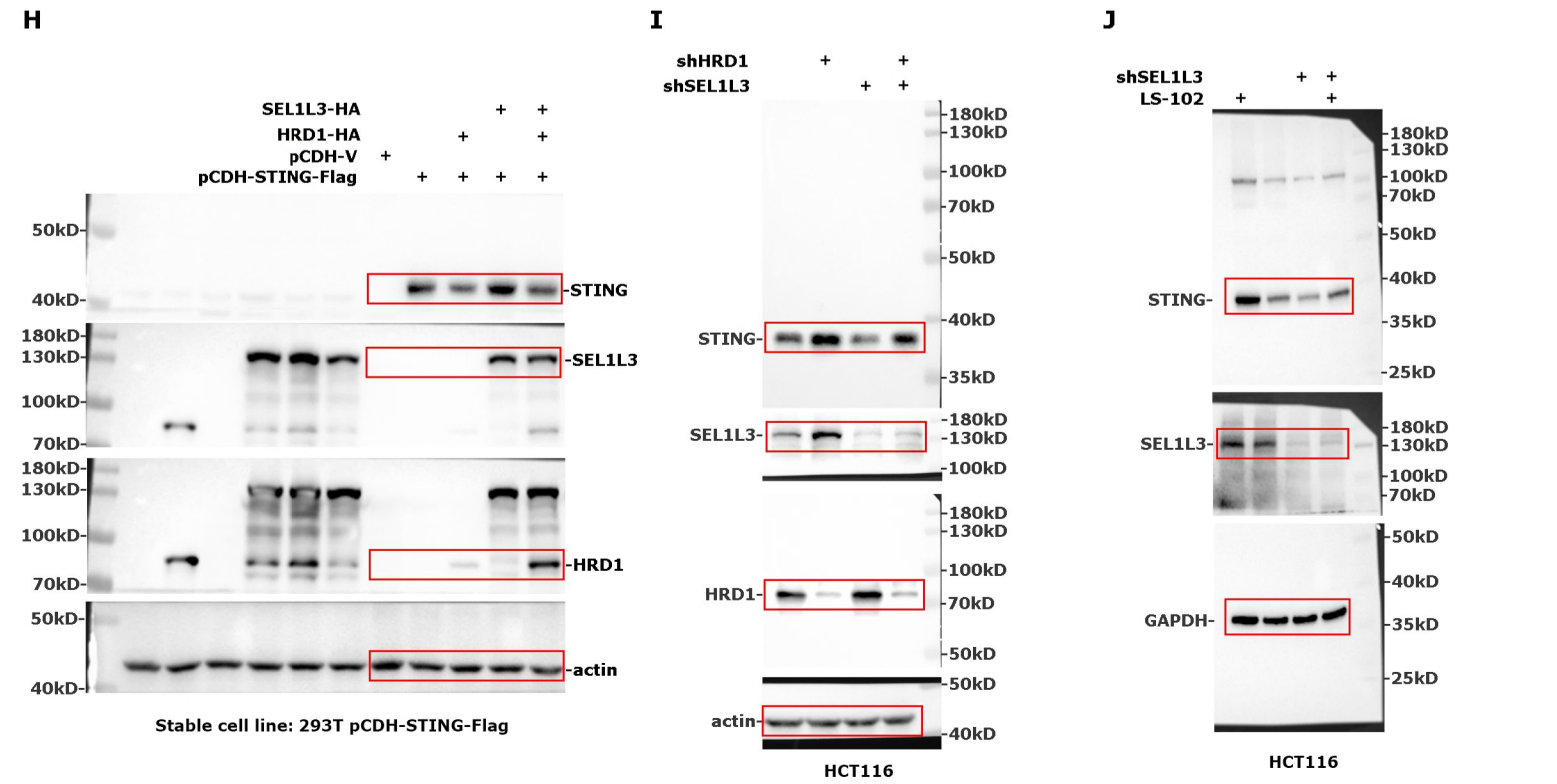

Figure 3

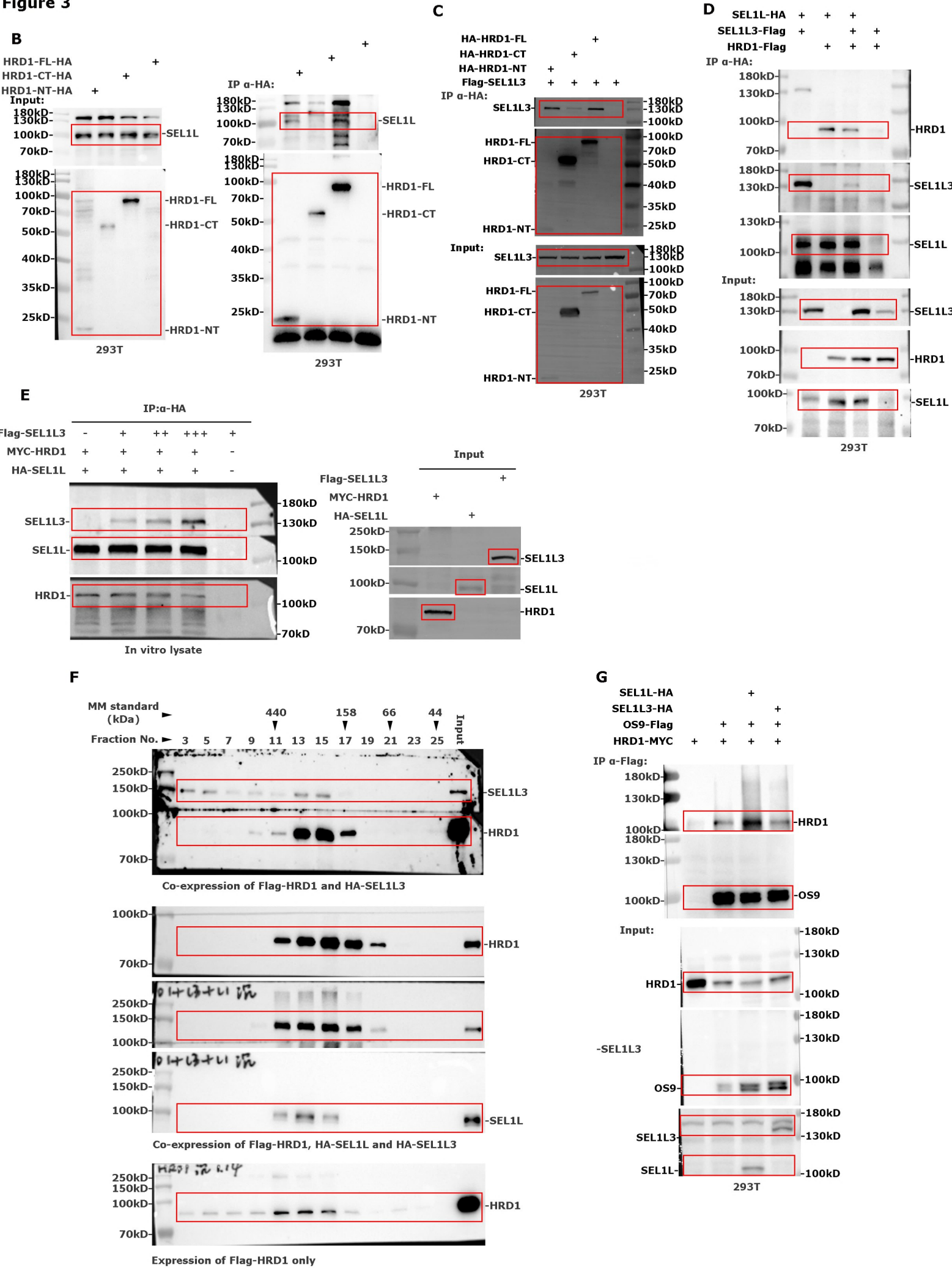

Figure 4

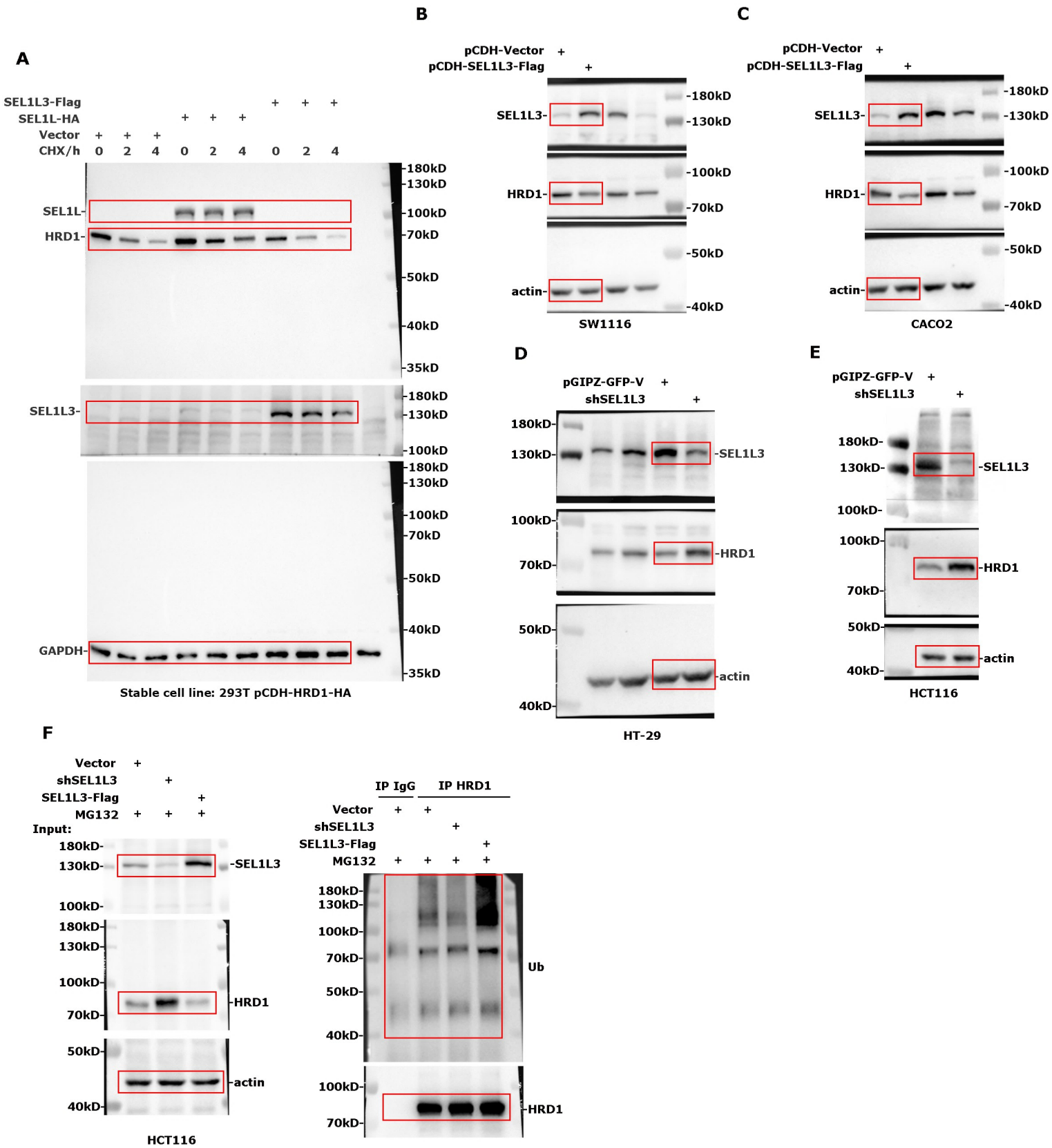

**Figure 5**

**A**

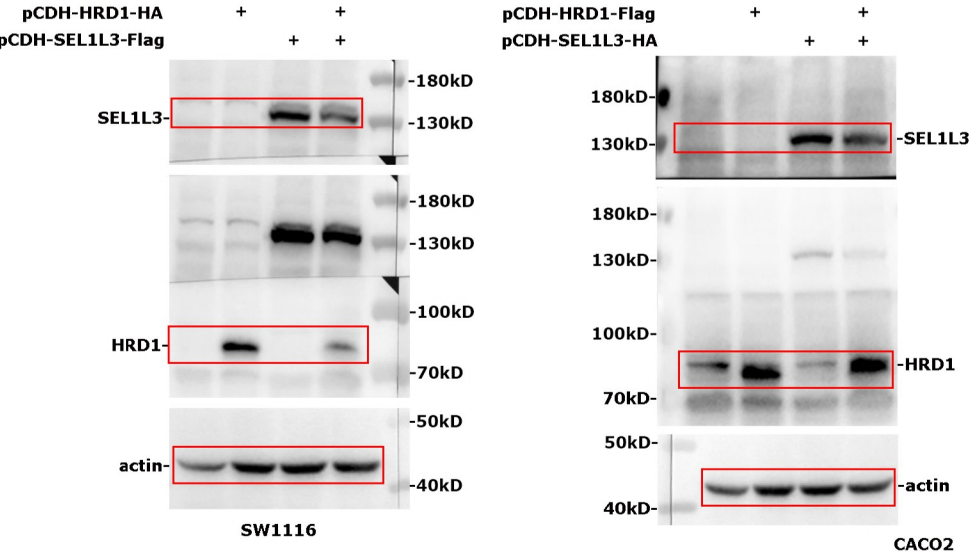

**Figure 6**

**A**

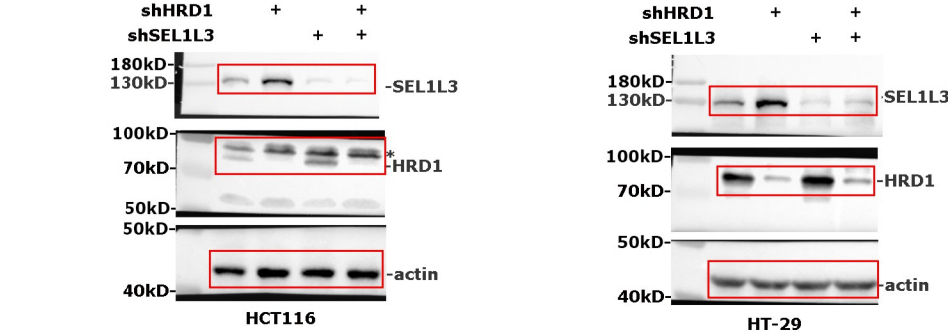

**Figure 7**

**A**

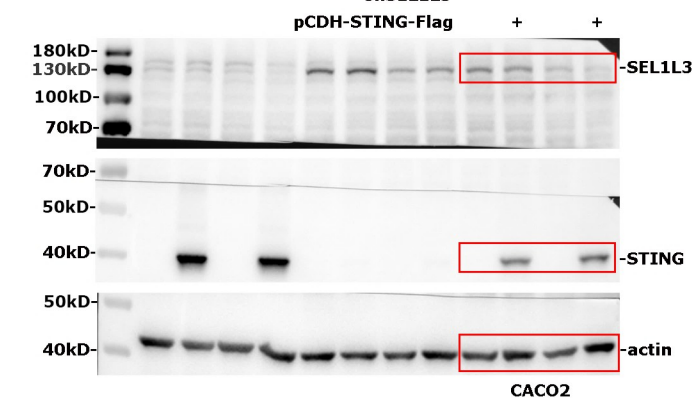

**F**

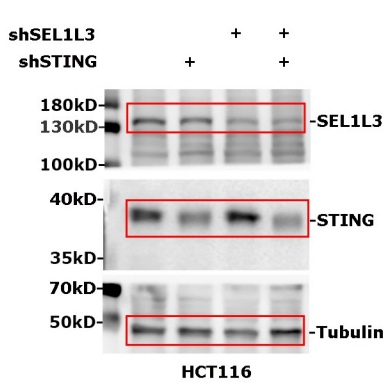

**Figure 8**

**A**

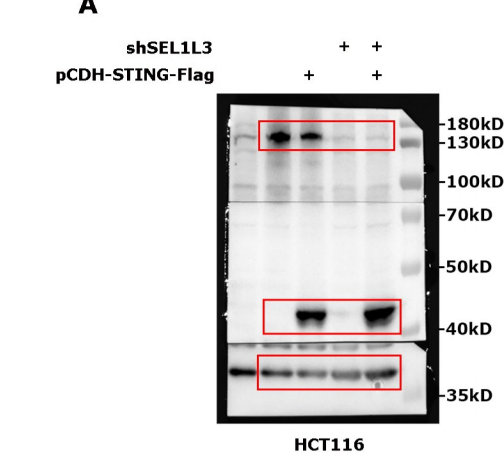

**Figure S1**

**A**

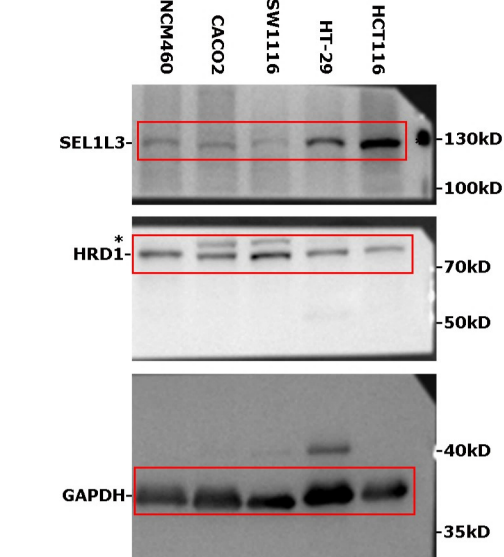

Supplement: Supplementary file 3 — Original Data [file 41419_2026_8770_MOESM3_ESM.pdf]
